# Oro-Dental Characteristics Associated with Pediatric Dental Neglect: A Retrospective Study

**DOI:** 10.3390/children12091266

**Published:** 2025-09-21

**Authors:** Anamaria Violeta Țuțuianu, Abel Emanuel Moca, Teodora Ștefănescu, Dan Alexandru Slăvescu, Lucian Roman Șipoș, Horia Câlniceanu, Anca Ionel

**Affiliations:** 1 Department of Dentistry, Faculty of Medicine and Pharmacy, University of Oradea, 10 Piața 1 Decembrie Street, 410073 Oradea, Romania; anamaria.tutuianu@uoradea.ro (A.V.Ț.); tstefanescu@uoradea.ro (T.Ș.); slavescudan@uoradea.ro (D.A.S.); lsipos@uoradea.ro (L.R.Ș.); 2 Department of Periodontology, Faculty of Dental Medicine, Anton Sculean Research Center for Periodontal and Peri-Implant Diseases, Victor Babeș University of Medicine and Pharmacy, 300041 Timișoara, Romania; calniceanu.horia@umft.ro; 3 Department of Oral Rehabilitation, Faculty of Dental Medicine, Iuliu Hațieganu University of Medicine and Pharmacy Cluj-Napoca, 400012 Cluj-Napoca, Romania; ionel.anca@umfcluj.ro

**Keywords:** dental neglect, pediatric oral health, socio-economic risk factors

## Abstract

**Background/Objectives:** Dental neglect represents a preventable form of child maltreatment that may lead to significant oral and systemic health complications. This study primarily aimed to investigate the main oral manifestations and determinants of dental neglect in a pediatric population from Bihor County, Romania. Additionally, it assessed the association between systemic factors, such as nutritional status and psychological conditions, and the severity of oro-dental lesions, with the goal of informing future preventive strategies and public health interventions. **Methods:** A retrospective analysis was conducted on 333 pediatric patients diagnosed with dental neglect. Clinical data were collected from two centers between 2020 and 2024. Oral health status, socio-demographic characteristics, and psychological conditions were evaluated. Statistical analyses included Fisher’s Exact Test, Mann–Whitney U Test, and Bonferroni-adjusted Z-tests, with significance set at *p* ≤ 0.05. **Results:** Among the 333 participants, 52.9% were male, with a mean age of 8.75 ± 3.29 years. Most children (81.7%) resided in rural areas, and 55.6% were first-born. Carious lesions were identified in 100% of cases, with 54.7% showing complications such as endodontic pathology. Poor oral hygiene was reported in 99.1% of children, while 58.6% presented signs of periodontal disease and 37.2% reported spontaneous gingival bleeding. Acute pain was experienced by 40.2% of participants. Nutritional issues were prevalent, with 24.3% classified as obese and 21.6% as malnourished. Significant associations were found between lesion severity and both psychological disorders (*p* < 0.001) and malnutrition (*p* < 0.001). **Conclusions:** This study identifies untreated carious lesions, poor oral hygiene, acute dental pain, and oro-dental trauma as key clinical indicators of pediatric dental neglect, with rural residency and limited education as significant socio-demographic risk factors. The findings highlight the urgent need for integrated, community-based strategies, including school-based screenings, parental education, and referral pathways, to improve early detection and prevention in Romania.

## 1. Introduction

Oral health represents a fundamental component of general health, particularly during childhood, a critical developmental stage when oral hygiene habits are formed, and permanent dentition emerges. Dental neglect can severely compromise children’s oral health, frequently resulting in a range of distinctive clinical manifestations [1]. These include an increased prevalence of dental caries, spontaneous gingival bleeding indicative of poor oral hygiene, acute dental pain, and unintentional dental trauma associated with advanced tissue degradation. Furthermore, children experiencing dental neglect often endure persistent pain, which may negatively affect their quality of life and hinder their overall developmental progress [1,2,3,4,5].

Dental neglect, classified as a form of maltreatment by omission, occurs when parents or caregivers fail to provide the necessary oral healthcare, leading to preventable dental conditions. According to the American Academy of Pediatric Dentistry (AAPD), “dental neglect is the willful failure of a parent or guardian to seek and follow through with treatment necessary to ensure a level of oral health essential for adequate function and freedom from pain and infection” [6]. This neglect impairs the child’s ability to maintain adequate oral function, potentially resulting in long-term health consequences. Moreover, the absence of timely and appropriate dental care can have far-reaching effects on the child’s general health, educational performance, communication abilities, psychosocial development, and self-esteem [1,6].

Preventing dental neglect requires the integration of comprehensive childcare strategies that promote nutritional support, encourage consistent oral hygiene practices, and ensure access to appropriate medical and dental services. Equally important is the education of caregivers regarding the significance of routine dental check-ups and early intervention to address emerging oral health issues. Facilitating access to these resources is essential for protecting both the oral and general health of children [1,3,7,8,9].

In Romania, pediatric dental services are theoretically covered by the national health insurance system, which allows children under 18 years of age to receive preventive and basic dental care free of charge. However, in practice, access to these services is uneven and often limited. Only dental practitioners who have a valid contract with the National Health Insurance House (NHIH) can provide reimbursed services, and even then, they operate under a strict monthly budget ceiling. Once this ceiling is reached, which often happens early in the month, no further public funding is available, and families must pay out-of-pocket. Additionally, the reimbursement rates set by the NHIH are smaller in value, discouraging many private providers from joining the system [10]. As a result, even though a basic structure for publicly funded care exists, it often fails to meet the needs of vulnerable populations, thereby contributing to systemic dental neglect.

The dentist plays a pivotal role in the detection and documentation of suspected child abuse or neglect; however, accurate identification remains a significant challenge. Clinicians must be trained to recognize specific intraoral and extraoral indicators of neglect, necessitating thorough clinical examinations [2,3,4,5]. In suspected cases of neglect, all dental, periodontal, and periapical findings should be meticulously recorded in the patient’s medical file, and an appropriate treatment plan should be developed and clearly communicated to the parents or caregivers [2,3].

The practitioner must ensure that caregivers fully understand the treatment plan, its importance in restoring the child’s oral health, and the necessity of attending follow-up appointments. Diagnostic documentation, including radiographs and clinical photographs, should be obtained during the initial visit to serve as evidence of existing oral lesions. Informed consent must be secured for data collection and photographic documentation, ensuring compliance with legal and ethical standards. Failure of parents or caregivers to adhere to scheduled dental appointments may further substantiate concerns of neglect [2,3,9]. In such cases, the dentist is obligated to report the suspected neglect to the appropriate child protection services or local law enforcement authorities.

In addition to oral manifestations, pediatric dental neglect is often accompanied by broader systemic consequences, such as nutritional imbalances and psychosocial disorders. Malnutrition and obesity, in particular, have been shown to correlate with poor oral health outcomes, and their presence may exacerbate the impact of dental neglect [2].

Given the increasing awareness of child maltreatment and its multifaceted health implications, dental neglect remains an underrecognized yet clinically significant concern, particularly in Central and Eastern Europe, where region-specific data are limited. Despite the known oral and systemic consequences of neglect, there is a lack of comprehensive studies in Romania that examine both clinical manifestations and associated psychosocial and nutritional factors [9].

Therefore, the present study primarily aimed to investigate the predominant oral manifestations associated with neglect and abuse in a pediatric population aged 4 to 17 years in Bihor County, Romania. In addition, it also assessed systemic health-related factors, such as nutritional status and psychological conditions, to better understand their association with the severity of oro-dental lesions. By identifying child-specific characteristics and determinants contributing to dental neglect, this study seeks to support future preventive strategies and targeted public health interventions.

## 2. Materials and Methods

### 2.1. Study Design and Ethical Considerations

This retrospective study was approved by the Research Ethics Committee of the Faculty of Medicine and Pharmacy within the University of Oradea (Approval No. CEFMF/08, dated 5 October 2020) and conducted in accordance with the ethical principles outlined in the 1964 Declaration of Helsinki and its subsequent amendments. All clinical data included in the study were collected from patients whose parents or legal guardians had provided written informed consent prior to their clinical assessment and documentation. Informed consent was obtained with particular sensitivity in cases where dental neglect was suspected, ensuring voluntariness and confidentiality, and separating research participation from clinical care.

### 2.2. Participants and Eligibility Criteria

The study population consisted of pediatric patients who attended consultations at the “Dr. Gavril Curteanu” Municipal Clinical Hospital—Pediatric Department and a private dental clinic in Oradea, Romania, over a four-year period (2020–2024).

Eligibility for inclusion was defined by the following criteria: children aged 4 to 17 years, residing in Bihor County, whose parents or legal guardians provided informed consent for clinical examination and photographic documentation. Only cases with oro-dental lesions consistent with dental neglect, as defined by the American Academy of Pediatric Dentistry [6], were included. Diagnosis was based on clinical indicators (e.g., untreated caries, poor oral hygiene, missed follow-ups) and corroborated by medical history and caregiver interviews.

Exclusion criteria comprising children with an uncertain diagnosis of dental neglect, children residing outside Bihor County, and cases where parental or guardian consent for participation was not obtained. Children with mental or general medical conditions were not excluded if their oral lesions were clinically consistent with neglect, as the study aimed to explore associations between systemic factors and dental neglect.

### 2.3. Clinical Assessment and Data Collection

The baseline evaluation included medical and dental anamnesis (conducted with caregiver assistance), a general clinical examination, and a full dental assessment comprising extraoral and intraoral inspection, supplemented by digital radiographs (Pax-i 3D Green, Vatech, Hwaseong, South Korea) and intraoral photographs (MD-1500A, Mouthwatch, Metuchen, NJ, USA). All examinations were performed by two calibrated dental practitioners (A.V.Ț. and A.E.M.) using a standardized diagnostic protocol, with findings recorded in structured forms. Radiographic and photographic records were reviewed jointly, and disagreements were resolved by consensus to minimize inter-observer variability.

The severity of oral lesions was pragmatically categorized into three groups: minor (non-complicated carious lesions without pulpal involvement or soft tissue inflammation, usually limited to one or two teeth), moderate (deep caries approaching or involving the pulp, early endodontic changes, or localized inflammation, typically affecting multiple teeth but without systemic complications), and severe (extensive destruction with pulp necrosis, abscesses, fistulas, or systemic manifestations such as fever or facial swelling). This categorization was adopted to ensure a pragmatic and clinically relevant stratification of disease severity, reflecting both local diagnostic practice and the need to capture the progression from uncomplicated caries to conditions with potential systemic involvement. By grouping cases into minor, moderate, and severe categories, we aimed to facilitate comparability across patients and highlight the spectrum of neglect-related oral health outcomes in a manner accessible to both clinical and public health perspectives.

Systemic and oral health variables were defined as follows:

- Oral hygiene—good if no visible plaque/calculus and healthy gingiva without bleeding; poor if plaque/calculus was present with gingival inflammation and/or bleeding [11].
- Obesity—BMI ≥ 95th percentile for age and sex according to WHO growth charts [12].
- Malnutrition—underweight appearance, delayed growth, or muscle wasting, and/or caregiver-reported undernutrition, consistent with WHO guidelines [13].
- Mental health status—assessed via caregiver report and clinical observation, categorized as (a) healthy, (b) mentally disabled, or (c) drug users. No formal psychological testing was performed.

Socio-demographic data included gender, living environment (urban/rural), child’s rank within the family, and the highest level of formal education at the time of clinical assessment.

The final study sample comprised 333 children diagnosed with dental neglect between 2020 and 2024. As a retrospective observational study, no formal sample size calculation was performed; all eligible cases identified during the study period were included.

### 2.4. Statistical Analysis

Statistical analysis was performed using IBM SPSS Statistics version 20 and Microsoft Office Excel/Word 2013. Data were first compiled into an Excel database, which was utilized for initial data management, including sorting and calculating descriptive statistics such as means and percentages. Microsoft Word 2013 was used for writing, formatting, and organizing the final manuscript.

Quantitative variables were tested for normality using the Shapiro–Wilk test and reported as means with standard deviations. Categorical variables were presented as absolute frequencies and percentages. Comparisons of independent quantitative variables were conducted using Student’s *t*-test for normally distributed data, and the Mann–Whitney U test or Kruskal–Wallis H test for non-normally distributed data. Correlations between variables were analyzed using Spearman’s rank correlation coefficient (Spearman’s rho).

Categorical data were analyzed using Fisher’s Exact Test, and Bonferroni-adjusted Z-tests were applied to refine the interpretation of qualitative variable comparisons. For post hoc analyses of independent quantitative variables, the Dunn-Bonferroni test was employed. Statistical significance was set at a *p*-value of ≤ 0.05.

## 3. Results

### 3.1. Socio-Demographic Characteristics of the Participants

A total of 333 children diagnosed with dental neglect were included. The mean age was 8.75 ± 3.29 years (median 8; range 4–17), and 52.9% were male. Most participants (81.7%) resided in rural areas. First-born children accounted for the largest proportion (55.6%), followed by second-born children. Regarding education, nearly half were enrolled in primary school (46.2%), while 7.2% had no formal education (Table 1).

**Figure 1:**
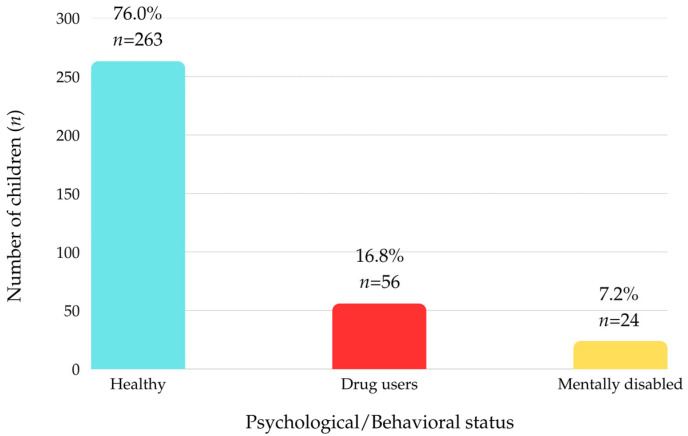
The psychological status of the subjects included in the study.

Psychological assessment showed that 7.2% of the participants had a diagnosed mental impairment, while 16.8% reported drug use, as illustrated in Figure 1.

### 3.2. Oro-Dental Characteristics of the Investigated Children

**Figure 2:**
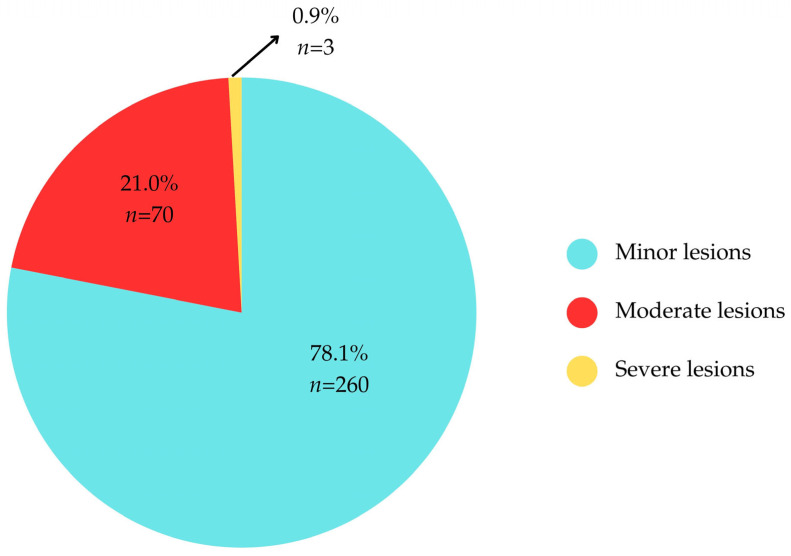
Distribution of children according to the severity of oro-dental lesions.

Analysis of the severity of oro-dental lesions among the study participants revealed that the majority of children (78.1%) presented with minor lesions, while a small proportion (0.9%) exhibited lesions classified as severe. These findings are illustrated in Figure 2.

Fisher’s Exact Test revealed statistically significant differences between the groups (*p* < 0.001). Post hoc analysis using Bonferroni-corrected Z-tests indicated that low-severity lesions were significantly more frequent among children without psychological or behavioral conditions (95.8% vs. 11.4%). In contrast, children with moderate (84.3% vs. 4.2%) or high-severity (4.3% vs. 0%) lesions were more commonly observed among those with diagnosed mental impairment or reported drug use. These results are detailed in Table 2.

Table 3 summarizes the clinical indicators of dental and oral health status among the study participants. Nearly all children (99.1%) exhibited poor oral hygiene, and caries was present in 100% of cases. More than half (54.7%) had lesions complicated by endodontic pathology, while 58.6% showed periodontal disease symptoms and 32.7% presented with spontaneous gingival bleeding. Acute pain at presentation was reported by 40.2% of children. Regarding nutritional status, 24.3% were classified as obese and 21.6% as malnourished.

Correlation analysis (Mann–Whitney U test) revealed significant associations between age and several clinical parameters. Children without psychological or behavioral conditions had a median age of 7 years (range 6–9), significantly lower than those with mental impairment or drug use (14 years, range 12–15; *p* < 0.001; data not included in the table). Similarly, children with focal infectious diseases were older (median 15 years vs. 8 years; *p* = 0.017). In contrast, those with unintentional dental trauma were younger (median 7 years, range 6–8) compared to children without trauma (12 years, range 11–15; *p* < 0.001).

### 3.3. Impact of Demographic, Nutritional, and Psychological Variables on Oral Health Status

Table 4 presents the distribution of children by gender and the severity of oro-dental lesions. Statistically significant differences were observed between groups, as indicated by Fisher’s Exact Test (*p* = 0.003). Post hoc Bonferroni-adjusted Z-tests revealed that low-severity lesions were more prevalent among female participants (85.4% vs. 71.6%), while moderate-severity lesions were more common among males (26.7% vs. 14.6%).

Children who were either mentally disabled or drug users had significantly poorer oral health outcomes than their healthy peers. All children in these categories exhibited poor oral hygiene, while good oral hygiene was observed only among healthy children (*p* = 0.047). Gingival bleeding was also more frequent among mentally disabled or drug-using children (68.6% vs. 23.2%; *p* < 0.001). Lesion severity further reflected this disparity: minor lesions were almost exclusive to healthy children (95.8%), whereas moderate (84.3%) and severe (4.3%) lesions occurred predominantly in children with mental disability or drug use (*p* < 0.001) (Table 5).

The severity of oro-dental lesions was also found to be associated with nutritional status. Fisher’s Exact Test showed that low-severity lesions were significantly more frequent among children without malnutrition (92.7% vs. 25%), whereas moderate (70.8% vs. 7.3%) and severe (4.2% vs. 0%) lesions were more frequently observed among malnourished children (*p* < 0.001) (Table 6).

Table 7 summarizes the distribution of children based on the severity of their oral lesions and their educational status. The Pearson Chi-Square test indicated statistically significant differences between groups (*p* < 0.001). Bonferroni-adjusted Z-tests demonstrated that children with no formal education were more likely to present with severe lesions (66.7%), while preschool-aged children had a higher prevalence of mild lesions (36.5%).

## 4. Discussion

While international bodies such as the American Academy of Pediatric Dentistry (AAPD) and the Royal College of Paediatrics and Child Health (RCPCH) have issued guidelines to support clinicians in identifying and managing dental neglect [4,6], the application of these recommendations varies widely across countries. In Romania, national oral health strategies have historically lacked focus on dental neglect as a clinical entity and detection remains reliant on clinicians’ individual vigilance [14]. This gap highlights the need for country-specific data and tailored strategies, such as those provided by the present study, which contributes evidence from a region where pediatric dental neglect remains poorly characterized in the literature. This study aimed to investigate the occurrence of dental neglect and associated oro-dental lesions in a pediatric population from both urban and rural areas of North-West Transylvania, Romania. We analyzed the relationships between the severity of oral lesions and participants’ general and mental health conditions, while also assessing how educational status relates to dental health and hygiene behaviors. To our knowledge, this is the first study conducted on a pediatric population in Romania that examines dental neglect and its clinical manifestations.

The gender distribution in our sample was relatively balanced, with males representing 52.9% and females 47.1%, showing a slight male predominance. The slightly higher proportion of males may reflect increased self-care awareness among adolescent girls, motivated by aesthetic concerns and earlier developmental milestones compared to boys. Previous research has consistently shown that adolescent girls tend to be more conscious of personal appearance and body image, often driven by aesthetic concerns and earlier developmental milestones compared to boys. For instance, surveys in the United Kingdom have revealed that nearly half of adolescent girls frequently worry about their body image, compared to only one-quarter of boys, suggesting a greater sensitivity to aesthetic ideals among females [15]. Similarly, studies on the influence of social media have highlighted that adolescent girls are more likely than boys to engage in appearance comparisons, which further intensifies their concern with self-image and motivates greater attention to self-care practices [16]. Our findings suggest that dental neglect is more strongly influenced by parental education and caregiving practices than by the child’s gender. The mean age of participants was 8.75 ± 3.29 years, with a median age of 8 years (range 4–17 years). These findings are consistent with reports from the literature, such as a 2015 study on child protection records concerning dental neglect, which reported a similar age distribution. Likewise, Zins et al., 2019, reported that 95% of children with dental neglect were under 10 years of age [17].

Our data indicate that first-born (55.6%) and second-born (32.7%) children were most frequently affected, with the majority (81.7%) residing in rural areas. Similarly to our study, Liu et al., 2021, reported that non-single children had poorer oral health-related quality of life (OHRQoL) and higher oral impacts on daily performance (OIDP), particularly in rural settings [18]. In comparison to our study, which highlighted the predominance of first and second-born children, their findings suggest that the presence of siblings itself may represent a risk factor, compounded by socioeconomic disadvantages in rural areas [18]. Contributing factors likely include parental inexperience, lack of education, limited awareness of long-term consequences, and socio-environmental barriers to healthcare access, which have also been identified in a recent study conducted in the same city (Oradea) addressing parental knowledge, attitudes, and practices regarding early childhood caries (ECC) [9]. However, this does not imply a deterministic pattern of neglect based on birth order; many parents, regardless of their age, socioeconomic status, or educational background, strive to provide appropriate care for their children. Social determinants such as family isolation, high numbers of children per household, unemployment, and low socioeconomic status, especially in rural areas, have been previously identified as risk factors for dental neglect [7,19]. Although some of these variables were not directly assessed in our study, they provide relevant context for interpreting our findings.

While most children in our study were otherwise healthy (79%), a concerning proportion were identified as drug users (16.8%) or as having intellectual disabilities (7.2%). Similarly to our findings, national-level data from the Romanian population report that approximately 19.2% of high-school students have experimented with illicit drugs or new psychoactive substances at least once [20]. Substance abuse, particularly among adolescents, may serve as a maladaptive coping mechanism or a means of seeking attention from caregivers. Such behaviors can mask symptoms of dental neglect, allowing oral lesions to progress unchecked. Regarding educational attainment, most children were either attending or had not completed primary education (46.2%), while 28.5% were in preschool and 7.2% had no formal education.

In our study, all participants presented with carious lesions affecting both primary and permanent teeth, with more than half showing complications related to endodontic pathology. Consistent with prior research, severe untreated caries have been reported as a characteristic feature of dental neglect [21]. Similar findings were reported in a U.S. study by Valencia-Rojas et al., where children identified as neglected had significantly higher dmft/DMFT scores and more extensive untreated caries compared to their counterparts [22]. Notably, a comprehensive review by Bradbury-Jones et al., 2013, emphasizes that children who experience broader forms of maltreatment have significantly higher levels of tooth decay. For example, five-year-olds with a documented history of maltreatment had nearly twice as many carious lesions as their non-maltreated counterparts, and in a U.S.-based cohort, neglected children between ages 5 and 13 were almost eight times more likely to have untreated, decayed permanent teeth [19].

Indicators such as plaque accumulation, calculus, gingival bleeding, and reports of acute dental pain further underscore the presence of neglect [21]. In our sample, 40.2% of children reported acute pain, 60.7% had unintentional trauma resulting from extensive carious destruction, and 54.7% presented with endodontic complications, including pulpitis, gangrene, and abscesses. These findings align with data from pediatric dental emergencies in Oradea: during the COVID-19 pandemic, pulpitis accounted for approximately 40.5% to 43.9% of emergencies, while acute apical periodontitis comprised around 42.6% [5]. Internationally, the prevalence of dental pain among children and adolescents averages about 36.2%, with up to 48.8% of emergency cases involving endodontic-related issues, including pulpitis and abscesses [23]. In regard to dental trauma, our results stand in stark contrast to global epidemiological benchmarks because systematic reviews indicate that dental trauma affects approximately 17.5% of children and adolescents worldwide, significantly lower than the 60.7% trauma rate in our sample. One comprehensive meta-analysis focusing on primary dentition reports a prevalence of 22.7% for dental trauma, again notably lower than what we observed [24]. Such a discrepancy strongly suggests that in our population, trauma is not merely the result of accidental injury, but is closely tied to untreated caries and, ultimately, to dental neglect. Progression of untreated caries to pulp involvement and apical pathology poses further health risks, particularly in permanent teeth. The progression of untreated caries to pulp involvement and apical pathology poses further health risks, particularly in permanent teeth. In rural Romania, limited availability of pediatric dental providers, long distances to care facilities, and lower parental health literacy contribute to significant delays in seeking treatment [25]. This is consistent with prior research showing that dental neglect is disproportionately more prevalent among children from rural areas compared to their urban peers [18].

Only 0.9% of participants demonstrated adequate oral hygiene, while 99.1% showed plaque accumulation, 58.6% presented with calculus and periodontal disease symptoms, and 32.7% exhibited spontaneous gingival bleeding. These findings align with previous studies linking poor oral hygiene to dental neglect. Spiller et al., 2020, identified plaque accumulation and gingival inflammation, such as spontaneous bleeding, as core clinical markers of dental neglect [1]. Similarly, studies among Chinese adolescents report gingival bleeding in 46.6% and calculus in 66.9%, both strongly associated with deficient oral health behaviors and low awareness [26].

Most children presented with mild lesions (78.1%), while 21% had moderate and 0.9% had severe lesions. Even mild untreated lesions pose risks of progression to more severe conditions, including sleep disturbances, social withdrawal, weight loss, developmental delays, and reduced quality of life. Chronic infections in primary teeth can affect the developing permanent dentition, increasing the risk of systemic health complications such as respiratory diseases, diabetes, and long-term oral health problems [27].

Statistical analysis revealed significant age-related differences in clinical and behavioral profiles. Male participants had a higher median age (8.5 years) compared to females (7 years) (*p* = 0.001). Children with mental impairments or drug use were significantly older (12–15 years, median 14) than their healthy counterparts (6–9 years, median 7) (*p* < 0.001). Children experiencing unintentional trauma due to severe carious lesions were younger (6–8 years), consistent with anatomical differences between primary and permanent teeth, including thinner enamel, larger pulp chambers, and reduced mineralization, which increase susceptibility to decay and structural damage [28]. Additionally, malnutrition was significantly associated with more severe dental lesions (*p* < 0.001).

Healthy children were predominantly female (84.7%), while drug use and health problems were more frequent among male participants (26.1%) (*p* = 0.016). Children from rural areas were more likely to have incomplete primary education (49.6% vs. 31.1%), while those from urban areas achieved higher levels of education, including incomplete and completed secondary education (*p* < 0.001). Children with no formal education had the highest prevalence of severe lesions (66.7%) compared to those with lower rates of mild (5.8%) or moderate (10%) lesions (*p* < 0.001). These findings emphasize the role of educational environments in promoting oral and general hygiene awareness, helping to mitigate the effects of parental neglect. Although symptom severity is indeed influenced by the child’s age [29], our analysis also considers educational level as a proxy for both age and health-related behavioral exposure.

Beyond physical and social determinants, the psychological consequences of dental neglect deserve closer examination. Neglected children frequently present with heightened dental fear and anxiety, which can lead to further avoidance of care and worsening oral health. A recent study by Kvesić et al., 2023, examined risk factors for dental anxiety in children with traumatic dental injuries, identifying pain, poor oral hygiene, and insufficient parental knowledge as key contributors. These factors overlap with the conditions observed in our sample and support the need for early caregiver education and emotional support for affected children [30]. Moreover, the association between psychological or behavioral disorders and poor oral health may be bidirectional. Children with mental impairment or substance use disorders are particularly vulnerable, as cognitive limitations or addictive behaviors may hinder self-care and delay help-seeking [31]. At the same time, untreated dental pain and visible oral damage can exacerbate social stigma and emotional distress, especially in school-age children, potentially reinforcing cycles of neglect and avoidance [32]. This underscores the importance of integrating dental care with mental health services, especially in underserved pediatric populations.

While this study provides valuable insights into the dimensions of dental neglect in children, several limitations should be acknowledged. First, the retrospective design, relying on clinical records and caregiver-reported information, may have introduced recall or reporting bias, potentially affecting the accuracy of psychosocial and behavioral data. Future prospective studies with standardized questionnaires and direct child assessments could help overcome this limitation. Second, the study sample was restricted to two centers in Bihor County, Romania, which may limit generalizability; broader, multicenter research would allow for more representative findings. The absence of longitudinal follow-up also prevented evaluation of long-term outcomes or the effectiveness of interventions, highlighting the need for cohort studies that track neglected children over time. Finally, the lack of standardized diagnostic indices for oral hygiene and periodontal status may have influenced the consistency of assessments; future studies would benefit from validated indices to facilitate comparability across populations. Despite these limitations, this study is, to our knowledge, the first to document the clinical patterns of dental neglect in Romanian children, providing important baseline data to guide future research and inform targeted public health strategies for early detection and prevention.

Moreover, the long-term consequences of untreated dental neglect warrant closer attention. Chronic oral infections during childhood, especially those involving pulp and periapical tissues, have been associated with an increased risk of systemic complications in adulthood, including cardiovascular disease, diabetes, and respiratory illnesses [33,34]. Additionally, persistent dental pain and visible damage may negatively impact school attendance, academic performance, and self-esteem, thereby reducing educational and social opportunities later in life [35]. These findings suggest that dental neglect in early life can set off a chain of adverse health, psychological, and socioeconomic outcomes, reinforcing the urgency of timely intervention and prevention strategies.

Beyond its contribution to the academic understanding of pediatric dental neglect, this study also holds practical relevance for clinical practice, public health policy, and dental education. Clinically, our findings underscore the importance of adopting a multidisciplinary approach when managing neglected children, incorporating not only dental treatment but also psychological screening, nutritional evaluation, and caregiver engagement. From a policy perspective, the high prevalence of untreated dental conditions among children from rural areas suggests the urgent need to improve access to oral healthcare and integrate oral health components into broader child welfare programs. These systemic barriers, compounded by socioeconomic vulnerability, may partially explain the higher burden of dental neglect observed in these communities. These findings may also support the development of national protocols that foster collaboration between dental professionals and child protection services, as well as the implementation of school-based screening and preventive dental programs aimed at early identification of at-risk children. Furthermore, these results highlight the value of incorporating targeted training on the recognition and management of dental neglect into undergraduate and postgraduate dental education, better equipping future professionals to identify and respond to complex cases in practice.

## 5. Conclusions

The findings of this study reinforce that dental caries, along with indicators such as poor oral hygiene, acute dental pain, and oro-dental trauma, are key clinical hallmarks of pediatric dental neglect. These manifestations are closely linked to systemic and psychosocial consequences, affecting not only the child’s oral and general health but also emotional well-being and development. Rural residency and limited educational attainment emerged as significant socio-demographic risk factors, pointing to structural inequities in healthcare access and awareness.

Addressing dental neglect requires more than clinical intervention. It calls for interdisciplinary collaboration among dental professionals, social workers, educators, and public health authorities. Practical measures, such as implementing school-based dental screenings, establishing clear referral pathways to child protection services, and offering parental education programs, can contribute to early detection and prevention.

This study provides foundational data for Romania and underscores the urgent need for integrated, community-based strategies to protect vulnerable children and promote equitable oral health outcomes.

## Figures and Tables

**Table 1:** The socio-demographic characteristics of participants.

Variable		No.	Percentage
Gender	Female	157	47.1%
	Male	176	52.9%
Environment	Rural	272	81.7%
	Urban	61	18.3%
Child’s Rank	First	185	55.6%
	Second	109	32.7%
	Third	34	10.3%
	Fourth	5	1.5%
Child’s Formal Education	Without studies	24	7.2%
	Preschool level	95	28.5%
	Primary studies not completed	154	46.2%
	Completed primary studies	26	7.8%
	Unfinished secondary school	18	5.4%
	Completed secondary school	9	2.7%
	Unfinished high school education	7	2.1%

**Table 2:** Distribution of children according to psychological status and severity of oro- and dental lesions.

Psychological Status/ Severity of Lesions	Drug User/ Mentally Disabled		Healthy		*p* *
	No.	Percentage	No.	Percentage	
Minor oro-dental lesions	8	11.4%	252	95.8%	<0.001
Moderate oro-dental lesions	59	84.3%	11	4.2%	
Severe oro-dental lesions	3	4.3%	0	0.0%	

* Fisher’s Exact Test.

**Table 3:** Participant distribution based on their oral-dental and general health status.

Variable		No.	Percentage
Good oral hygiene	Yes	3	0.9%
	No	330	99.1%
Spontaneous gingival bleeding	Yes	109	32.7%
	No	224	67.3%
Caries lesions	Yes	333	100.0%
	No	0	0.0%
Caries complicated by endodontic pathologies	Yes	182	54.7%
	No	151	45.3%
Unintentional trauma	Yes	232	69.7%
	No	101	30.3%
Acute pain	Yes	134	40.2%
	No	199	59.8%
Periodontal disease symptoms	Yes	195	58.6%
	No	138	41.4%
Obesity	Yes	252	24.3%
	No	81	75.7%
Malnutrition	Yes	72	21.6%
	No	261	78.4%
Focal infection disease	Yes	3	0.9%
	No	330	99.1%

**Table 4:** Distribution of children by gender and oro-dental lesion severity.

Gender/ Severity of Lesions	Female		Male		*p* *
	No.	Percentage	No.	Percentage	
Minor oro-dental lesions	134	85.4%	126	71.6%	0.003
Moderate oro-dental lesions	23	14.6%	47	26.7%	
Severe oro-dental lesions	0	0.0%	3	1.7%	

* Fisher’s Exact Test.

**Table 5:** Oral health characteristics in children according to their psychological status.

Psychological Status	Drug User/ Mentally Disabled		Healthy		*p* *
	No.	Percentage	No.	Percentage	
Oral hygiene					
Good oral hygiene	0	0.0%	15	5.7%	0.047
Poor oral hygiene	70	100.0%	248	94.3%	
Spontaneous gingival bleeding					
No spontaneous bleeding	22	31.4%	202	76.8%	<0.001
With spontaneous bleeding	48	68.6%	61	23.2%	
Severity of oro-dental lesions					
Minor lesions	8	11.4%	252	95.8%	<0.001
Moderate lesions	59	84.3%	11	4.2%	
Severe lesions	3	4.3%	0	0.0%	

* Fisher’s Exact Test.

**Table 6:** Severity of oro-dental lesions in relation to malnutrition.

Malnutrition/ Severity of Lesions	Without Malnutrition		With Malnutrition		*p* *
	No.	Percentage	No.	Percentage	
Minor lesions	242	92.7%	18	25.0%	<0.001
Moderate lesions	19	7.3%	51	70.8%	
Severe lesions	0	0.0%	3	4.2%	

* Fisher’s Exact Test.

**Table 7:** Distribution of children according to the severity of oro-dental lesions and educational level.

Severity of Lesions/Child’s Formal Education	Minor Oro-Dental Lesions		Moderate Oro-Dental Lesions		Severe Oro-Dental Lesions		*p* *
	No.	Percentage	No.	Percentage	No.	Percentage	
Without studies	15	5.8%	7	10.0%	2	66.7%	<0.001
Preschool level	95	36.5%	0	0.0%	0	0.0%	
Primary studies not completed	117	45.0%	37	52.9%	0	0.0%	
Completed primary studies	18	6.9%	8	11.4%	0	0.0%	
Unfinished secondary school	7	2.7%	10	14.3%	1	33.3%	
Completed secondary school	6	2.3%	3	4.3%	0	0.0%	
Unfinished high school education	2	0.8%	5	7.1%	0	0.0%	

* Pearson Chi-Square Test.

## Data Availability

The data presented in this study are available on request from the corresponding authors. The data are not publicly available due to privacy reasons.
